# High prevalence of MRSA and multi-resistant gram-negative bacteria in refugees admitted to the hospital—But no hint of transmission

**DOI:** 10.1371/journal.pone.0198103

**Published:** 2018-05-31

**Authors:** Annelene Kossow, Bianca Stühmer, Frieder Schaumburg, Karsten Becker, Birgit Glatz, Mareike Möllers, Stefanie Kampmeier, Alexander Mellmann

**Affiliations:** 1 Institute of Hygiene, University Hospital Münster, Münster, Germany; 2 International Patient Management, University Hospital Münster, Münster, Germany; 3 Institute of Medical Microbiology, University Hospital Münster, Münster, Germany; 4 Clinic for Gynecology and Obstetrics, University Hospital Münster, Münster, Germany; Ross University School of Veterinary Medicine, SAINT KITTS AND NEVIS

## Abstract

With high numbers of refugees arriving in Europe uncertainty exists as to whether multidrug-resistant organisms are imported into the healthcare system. In our study, we identified 383 refugee-inpatients admitted to the University Hospital Münster, Germany between September 2015 and September 2016. For this patient cohort screening for Methicillin-resistant *Staphylococcus aureus* (MRSA), multidrug-resistant Gram-negative bacteria (MDR-GNB) and Vancomycin-resistant enterococci (VRE) was recommended in our institution. Until May 2016 pre-emptive isolation was applied to all refugee-inpatients until the exclusion of these multidrug-resistant organisms. MRSA were found in 34 refugee-patients (9.8%), MDR-GNB in 25 refugee-patients (12.9%) and VRE in none of the refugee patients. We did not find any strains carrying carbapenemases. Whole genome sequencing (WGS) data demonstrated that the respective isolates were genetically heterogeneous and revealed no transmission of refugee-patient isolates to other patients. We therefore omitted pre-emptive isolation as an infection control measure for this group of patients. Furthermore, molecular typing did not show evidence for nosocomial transmission from refugee-patients to other patients. Standard hygiene measures successfully prevented the transmission of refugee-patient isolates to other patients and as a result introduction into the healthcare system. This underlines that any multidrug-resistant organisms present within this cohort are not of any extraordinary concern for health systems.

## Introduction

Europe and Germany in particular experienced a dramatic rise of refugees seeking asylum during the past years [1]. A total of 441.899 people filed an asylum request in 2015. The Main countries of origin were Syria, Iraq and Afghanistan followed by Albania, Kosovo and Serbia. It is assumed that 800.000 to 1 million refugees were registered in Germany in 2015 [1].

Hospitals are challenged by the medical care of refugees. Language barriers and intercultural difficulties are add to the challenge of their increased need for medical care [2, 3]. Infection prevention measures are of special importance since several countries or regions of origin are known for a comparatively high prevalence of multidrug-resistant organisms (MDRO) [4, 5]. Additionally it can be assumed that the poor sanitary and hygiene conditions during their travels led to a higher transmission of bacteria. Data about the health of refugees and the prevalence of MDRO are scarce [6]. For this reason, the University Hospital in Münster decided in September 2015 to treat inpatient refugees in contact isolation until the carrier status for methicillin-resistant *Staphylococcus aureus* (MRSA), multidrug-resistant Gram-negative bacteria (MDR-GNB), and vancomycin-resistant enterococci (VRE) was known. Initial studies revealed a high prevalence of MDRO in inpatient refugees [7–9]. Based on these findings, there was an ongoing discussion about the adequate infection control measures regarding refugee inpatients. Until today, it is not known whether these strains will actually enter the German health care system or not. We therefore assessed the occurrence of MDRO in refugee inpatients in our institution. To investigate whether these strains enter our institution, we compared whole-genome sequencing (WGS) data of most of the MDRO found in refugee-patients (RP) with those strains derived from non-refugee patients (NRP). These findings contribute to the discussion regarding infection control measures.

## Methods

### Setting

The University Hospital Münster (UKM) is a tertiary care facility with 1,457 beds. In total, 56,751 inpatient cases and 511,793 outpatient cases were treated in 2015. The catchment area consists of up to 5 million people in northwest Germany.

In March 2016, 4,064 refugees lived in the municipal facilities in the city of Münster, among them about 1,700 children. 1,636 spots were available in shelters (oral communication, city of Münster).

Patients with a refugee background (a stay in Germany of less than 15 months) were registered with a special indicator in the medical information system. The study included all RP that were treated as inpatients between September 2015 and September 2016. Psychosomatic and psychiatric patients as well as accompanying persons and newborns born at our institution did not undergo screening for MDRO. Readmissions were only screened if the previous hospital stay was dated back more than four weeks. If a MDRO was found in clinical specimens from outpatients, the isolate was included in the analysis. If the screening was positive, we further researched epidemiological data as age and countries of origin. One patient was excluded due to her unclear refugee status.

From September 2015 to May 2016 all patients identified as refugees were preemptively isolated. After the analysis of surveillance data, we omitted preemptive isolation from May 2016, but continued screening for MDRO.

NRP were screened for MRSA in the general admission screening established in our institution. Screening for MDR-GNB and VRE was performed in NRP if risk factors were present. NRP did therefore not serve as a systematic control group for prevalence of MDR-GNB and VRE.

### Ethical consideration

The screening was done based on the recommendation of the commission for infection prevention and control of the UKM and in accordance with the recommendations of the legally assigned institute for infection control and prevention (Robert Koch Institute). Therefore, an informed consent was not required. All patients are treated equally and in conditions of best medical care at UKM. All patient data was processed anonymized.

### Swabs and isolation practices

RP were screened for MDRO upon admission. Screening swabs (Transwab, medical wire, Corsham, UK) were taken from nose/throat and axilla/inguinal for MRSA, from anus and throat for MDR-GNB and from anus for VRE. Patients were placed in contact isolation until the results of the swabs were available and kept in contact isolation if swabs were positive for MRSA, MDR-GNB and/or VRE. If resistance in MDR-GNB was limited to 3 of 4 bactericidal classes contact isolation was ended.

### Microbiology

Detection of MRSA, MDR-GNB and VRE was carried out by using selective agar plates (chromID, BioMérieux, Marcy l’Etoile, France for MRSA, CHROMagar Acinetobacter, Mast Diagnostica, Reinfeld, Germany for *Acinetobacter* and Pseudomonas-Cetrimide, Oxoid, Wesel, Germany for *Pseudomonas aeruginosa*, *chromID ESBL* BioMérieux, Marcy l’Etoile, France and McConkey for MDR-GNB, VRE Select, Bio-Rad, Mames-la-Coguette, France for VRE) which were incubated at 36°C for 24 h or 48 h (Pseudomonas-Cetrimid and VRE). MRSA swabs were enriched in Dextrose Bouillon (24 h, 36°C) before being plated again on selective agar.

For species identification, we used MALDI-TOF mass spectrometry (Bruker, Daltonics, Bremen, Germany) or, if *E*. *coli* was suspected on the agar plate based on the phenotype, the VITEK-2 system (BioMérieux, Marcy l’Etoile, France). Resistances were tested using the VITEK-2 system for all species. MDR-GNB were categorized according to German national guidelines into MDR-GNB resistant to 3 or 4 bactericidal classes of antibiotics (Piperacillin, 3^rd^ generation cephalosporine, ciprofloxacin, carbapenem) [10, 11]. In the case of MRSA, resistances were confirmed by PBP2a (PBP2a SA Culture Colony Test, Scarborough, Maine, USA) and detection of the *mecA* and *mecC* genes, respectively (GenoType MRSA, HAIN, Nehren, Germany) [12, 13]. Resistance to vancomycin was confirmed by the detection of *van*A and *van*B, respectively (GenoType Enterococcus, HAIN, Nehren, Germany). Furthermore all RP isolates as well as isolates from NRP found in clinical specimens and/or screening were analyzed by WGS to detect possible genetic relationships amongst isolates. WGS was applied to one isolated per patient and species.

We used WGS as part of our routine surveillance strategy to determine possible clonal relationships between isolates derived from RP and isolates derived from NRP. DNA extraction, WGS library preparation, sequencing and subsequent data analysis were performed as recently described [14]. Using the SeqSphere^+^ software version 2 (Ridom GmbH, Muenster, Germany), we compared coding regions in a gene-by-gene approach (core genome multilocus sequence typing = cgMLST). Isolates from RP were compared with NRP-isolates derived up to January 2017 to investigate whether MDRO are brought into the health care setting by the admission of refugee patients. Isolates with a difference of <6 alleles for MRSA [15], <10 for *Escherichia coli* (*E*.*coli*) [16], <14 for *Pseudomonas aeruginosa* (P. *aeruginosa*), <8 for VRE [17], <15 for *Klebsiella pneumoniae* (*K*. *pneumoniae*) [18] were defined as being related. Clonal relationship was depicted in minimum-spanning trees (MST) generated by the SeqSphere^+^ software. Isolates with an allelic difference below the thresholds underwent epidemiological investigation for possible nosocomial transmission. Transmission was assumed if the patients corresponding to the isolates were treated at the same or related wards and isolates were detected within the period of one month. MLST sequence types (ST) and *spa* types were extracted from the WGS data *in silico* for backwards compatibility and assignment into clonal groups. Raw reads are deposited at the European Nucleotide Archive (ENA) under study accession number PRJEB23208.

### Statistics

We compared the prevalence of MRSA in RP with the prevalence in NRP that are generally screened upon admission at the UKM. We performed statistical analyses with Chi squared test with Yate’s correction. Results were considered statistically significant at a p-value <0.05.

## Results

During the study period, 606 refugees were treated as inpatients in our institution resulting in 383 cases that met the inclusion criteria to be recommended for screening for MDRO. The excluded 224 cases were in majority readmitted patients in addition to newborn babies born in our institution and psychiatric or psychosomatic patients and therefore not screened for MDRO according to infection control standards in our institution.

In the study period, 44,751 individual NRP were screened for MRSA in the general admission screening.

### Prevalence

In total, 587 of the 44,751 NRP screened in the general admission screening were positive for MRSA (1.4%).

Compliance to screening recommendations was variable resulting in 347 refugees being screened for MRSA, 225 refugees for MDR-GNB and 194 refugees for VRE, respectively. MRSA were found in 34 (9.8%), MDR-GNB in 25 (12.9%) and VRE in none of the surveillance cultures. One patient carried two species of MDR-GNB.

Statistical analyses of prevalence showed a significant difference in MRSA colonization between RP (9.8%) and NRP (1.4%) (OR 13.4; 95% CI: 9.0–19.7; p <0.001).

The screening for MDR-GNB revealed no occurrence of 4MRGN. We found 20 *E*. *coli*, three *K*. *pneumoniae* as well as one detection each of *P*. *mirabilis*, *P aeruginosa* and *M*. *morganii*. Carpabenemases harbouring strains were not detected.

Epidemiological data can be found in Table 1. In total 55 RP (median age 5 years (mean 15 years), range 0–64 years, 52.7% female) with carriage of MDRO (30 MRSA, 4 MRSA+MDR-GNB, 21 MDR-GNB) were detected during the observation period. The most common countries of origin were Syria (n = 16), Iraq (n = 10), and Afghanistan (n = 3). For 27 RP no information was available about the country of origin.

**Table 1:** Epidemiological data of refugee-patients (n = 56) carrying multidrug-resistant organisms, Münster, Germany September2015-September 2016.

Patient	No in Minimum spanning trees	Age (Years)	Country of origin	Sex	Pathogen	*spa*-Type^a^ and/or Sequence-Type^b^ (Clonal Cluster)
**MRSA**						
**1**	020	3	n.a.	f	MRSA	t127; ST1
**2**	018	35	Syria	m	MRSA	t127; ST1
**3**	011	2	Armenia	f	MRSA	t223; ST22
**4**	024	27	Syria	f	MRSA	t223; ST22
**5**	016	4	n.a.	f	MRSA	t012; ST30
**6**	041	4	Afghanistan	f	MRSA	t790; ST22
**7**	042	30	n.a.	f	MRSA	T690; ST88
**8**	044	5	Iraq	m	MRSA	T1627; ST6
**9**	050	55	Syria	m	MRSA	T034; ST398
**10**	056	19	n.a.	f	MRSA	t127; ST1
**11**	063	0,2	Iraq	f	MRSA	t304; ST6
**12**	065	27	Ghana	f	MRSA	t311; ST5
**13**	066	1	Iraq	m	MRSA	t304; ST6
**14**	012	8	Syria	f	MRSA	t991; ST913
**15**	015	32	Iraq	m	MRSA	t044; ST n.a.
**16**	083	6	n.a.	m	MRSA	t304; ST6
**17**	062	45	n.a.	m	MRSA	t5634; ST22
**18**	067	30	n.a.	f	MRSA	t223; ST22
**19**	087	0	n.a.	f	MRSA	t223; ST22
**20**	097	14	n.a.	m	MRSA	t363; ST30
**21**	096	26	n.a.	f	MRSA	t579; ST2626
**22**	119	61	n.a.	m	MRSA	t10740; ST859
**23**	118	21	n.a.	m	MRSA	t379; ST22
**24**	126	0	n.a.	m	MRSA	t127; ST1
**25**	134	23	n.a.	m	MRSA	t790; ST22
**26**	103	0	n.a.	m	MRSA	t5475; ST97
**27**	133	34	n.a.	f	MRSA	t690; ST88
**28**	145	5	Afghanistan	m	MRSA	t037; ST239
**29**	149	0	n.a.	f	MRSA	t620; ST45
**30**	032	4	n.a.	m	MRSA	t015; ST45
**31**	071	0	Syria	f	MRSA	t223; ST22
**32**	034	22	Syria	f	MRSA	t127; ST1
**33**	035	2	Syria	f	MRSA	t4573; ST22
**34**	108	25	n.a.	f	MRSA	t318; ST30
**MDR-GNB**						
**31**	072	0	Syria	f	*E*. *coli*	ST405 (CC405)
**32**	047	22	Syria	f	*E*. *coli*	ST131 (CC131)
**33**	036	2	Syria	f	*E*. *coli*	ST3873
**34**	110	25	n.a.	f	*E*. *coli*	ST131 (CC131)
**35**	017	2	Syria/Turkey	m	*E*. *coli*	ST131 (CC131)
**36**	014	24	n.a.	f	*E*. *coli*	ST6338
**37**	026	2	Syria	f	*E*. *coli*	ST131 (CC131)
**38**	025	0	Afghanistan	m	*E*. *coli*	ST131 (CC131)
**39**	033	29	n.a.	m	*K*. *pneumoniae*	ST16
**40**	029	5	Iraq	f	*E*. *coli*	ST10 (CC10)
**41**	037	0	Syria	m	*E*. *coli*	ST6335
**42**	045	31	n.a.	m	*E*.*coli*	ST405 (CC405)
**43**	040	64	Iraq	m	*E*.*coli*	ST131 (CC131)
**44**	023	4	Syria	m	*E*.*coli*	ST10 (CC10)
**45**	038	0	Syria	f	*E*. *coli*	ST131 (CC131)
**45**	039	0	Syria	f	*K*. *pneumoniae*	ST397
**46**		17	Iraq	m	*P*. *mirabilis*	n.a.
**47**	054	42	Pakistan	m	*E*. *coli*	ST648 (CC648)
**48**		5	Azerbaidschan	m	*P*. *aeruginosa*	ST385
**49**		1	Iraq/India	f	*E*. *coli*	n.s.
**50**	064	26	Iran	f	*E*. *coli*	ST 14 (CC14)
**51**		2	Iraq	m	*M*. *morganii*	n.a.
**52**	075	1	Iraq	m	*K*. *pneumoniae*	ST138
**53**	113	16	n.a.	f	*E*. *coli*	ST131 (CC131)
**54**	125	0	n.a.	f	*E*. *coli*	ST648 (CC648)
**55**	131	3	n.a.	f	*E*. *coli*	ST405 (CC405)

MRSA: Methicillin resistant *Staphylococcus aureus*, MDR-GNB: Multidrug resistant Gram negative bacteria, f: female, m: male, n.a.: not available, n.s.: not sequenced

^a^ For MRSA *spa*-Types are indicated.

^b^ If possible Sequence-types and clonal clusters are shown for all isolates

### Whole genome sequencing

**Fig 1:**
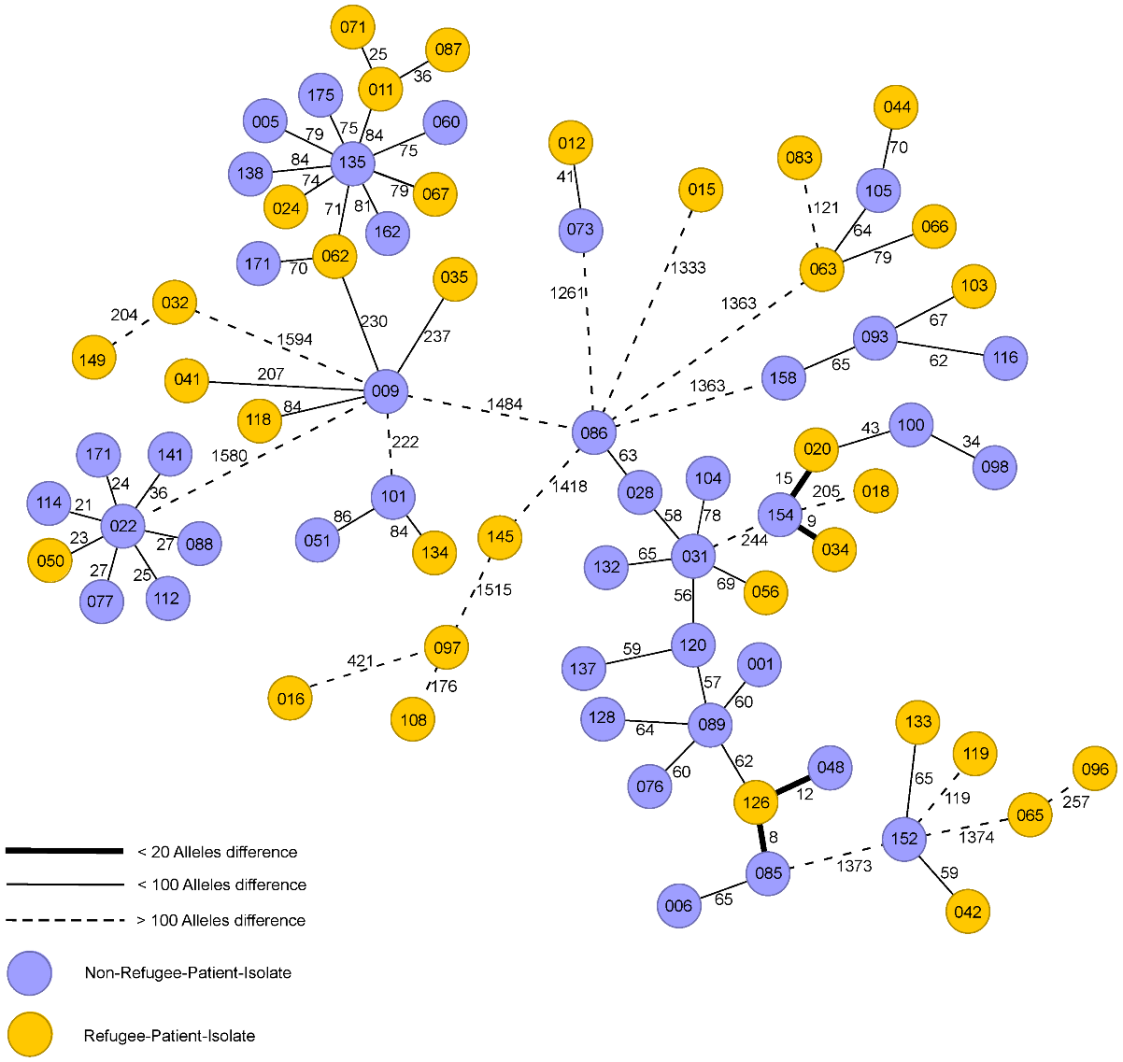
Minimum spanning tree for Methicillin resistant *Staphylococcus aureus* for 34 isolates from refugee patients. Isolates from refugee patients (yellow circles) from September 2015-September 2016 and similar samples from non-refugee-patients (n = 40, violet circles). Each circle representing a unique allele profile based on 1861 cgMLST target genes in the isolates with the “pairwise ignoring missing values” option turned on in the SeqSphere^+^ software during comparison. The thickness on connecting lines (not to scale) displaying the number of differing alleles between the genotypes. Circles are numbered according to the ascending order of date of collection with one month including 10 numbers on average.

**Fig 2:**
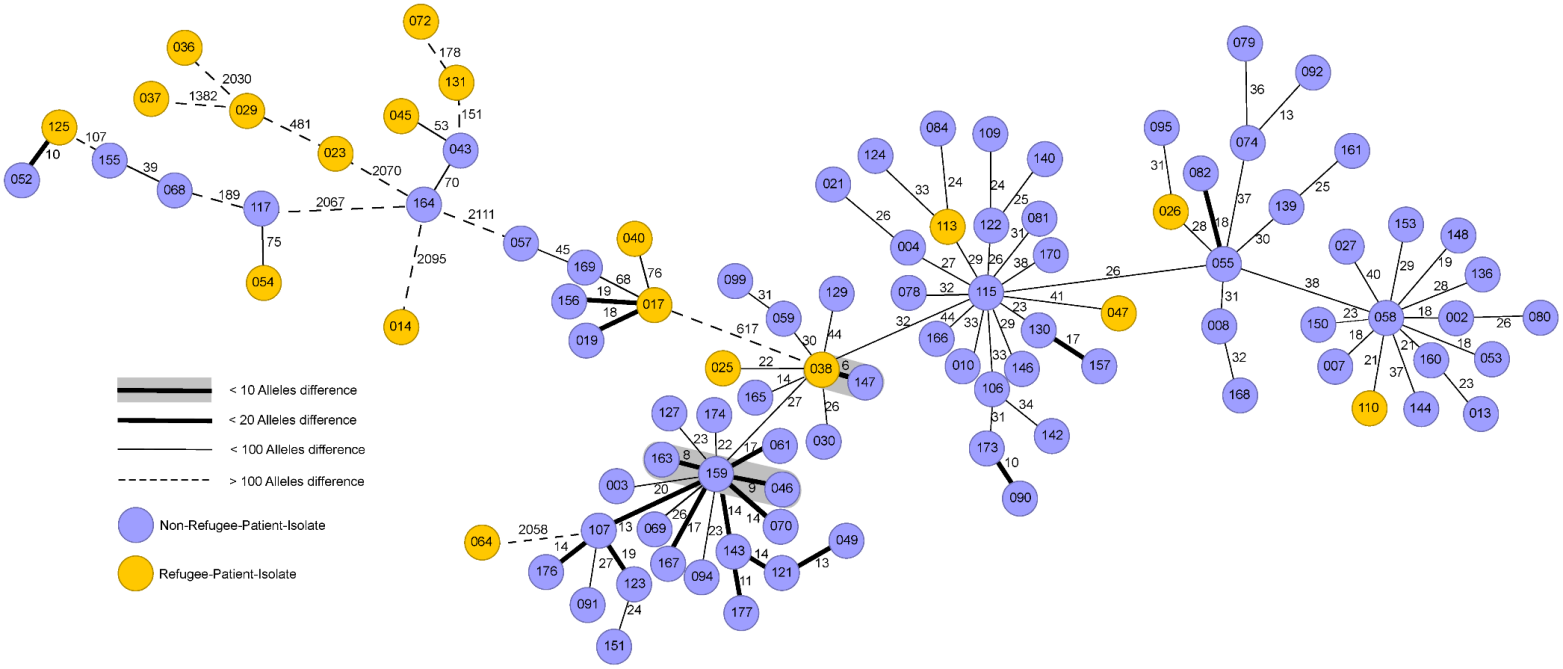
Minimum spanning tree for *Escherichia coli* for 19 isolates from refugee patients. Isolates from refugee patients (yellow circles) from September 2015-September 2016 and similar samples from non-refugee-patients (n = 78, violet circles). Each circle representing a unique allele profile based on up to 2325 cgMLST target genes in the isolates with the “pairwise ignoring missing values” option turned on in the SeqSphere^+^ software during comparison. The thickness on connecting lines (not to scale) displaying the number of differing alleles between the genotypes. Circles are numbered according to the ascending order of date of collection with one month including 10 numbers on average.

**Fig 3:**
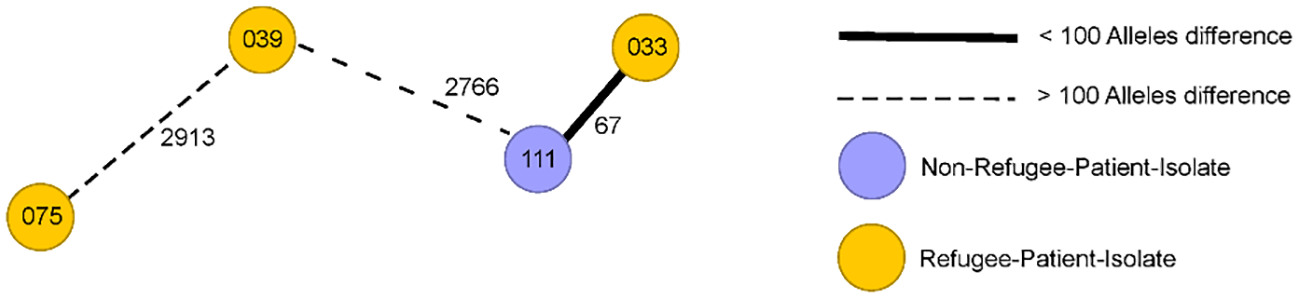
Minimum spanning tree *for Klebsiella pneumo*nia for three3 isolates from refugee patients. Isolates from refugee patients (yellow circles) from September 2015-September 2016 and similar samples from non-refugee-patients (n = 1, violet circles). Each circle representing a unique allele profile based on up 3220 cgMLST target genes in the isolates with the “pairwise ignoring missing values” option turned on in the SeqSphere^+^ software during comparison. The thickness on connecting lines (not to scale) displaying the number of differing alleles between the genotypes. Circles are numbered according to the ascending order of date of collection with one month including 10 numbers on average.

WGS was performed for all MDRO occurring in our institution. The results are displayed in a MST. If the number of alleles difference was lower than the defined threshold, in depth epidemiological investigation ruled out any nosocomial transmission. Analysis of MST did not reveal any clusters of RP-isolates up to five month after the study period. Figs 1–3 show MSTs for MRSA (Fig 1), *E*.*coli* (Fig 2) and *K*. *pneumoniae* (Fig 3).

For all other species, MSTs could not be generated due to a lack of similar isolates. In only one case, a close relationship was seen between an *E*. *coli* isolate from a RP and a later isolated *E*. *coli* from a corresponding NRP at the same ward. Since the later isolate occurred more than three months after the RP-isolate it was not rated as a nosocomial transmission. Therefore the analysis of WGS combined with epidemiological data showed that refugee patient isolates were not transmitted to any other patient treated at our institution.

The 34 MRSA isolates were associated with 14 different *spa* types (MLST ST): The most common *spa* types were t127 (ST1 n = 5; 14.7%), t223 (ST22 n = 5; 14,7%), t304 (ST6; n = 3, 8.8%), t690 (ST88; n = 2, 5.9%), t790 (ST22; n = 2, (5.9%). All other *spa* types can be found in Table 1 The main *spa* types (ST) in NRP were t032 (ST22; 18.4%), t003 (ST225; 17.2%), t034 (ST398; 15.2%), t011 (ST398; 12.0%), t127 (ST1; 6.2%), t002 (ST5, ST149, ST2626; 3.4%), t2011 (ST 398; 2.4%), t223 (ST22; 2.4%), t008 (ST8; 2.2%).

*E*. *coli* and *K*. *pneumonia* MLST STs of RP are displayed in Table 1.

## Discussion

With the rise of refugees seeking asylum in Europe, a debate started whether the medical treatment of this group of people requires special infection control measures based on the assumption of high prevalence of MDRO in this patient cohort. Whereas several studies found the prevalence of MDRO to be high in refugee-patients [7–9], the respective national German health agency (RKI) recommended not to take any special measures for refugee-patients [19]. In this study, we elucidated whether MDRO were actually introduced into the health system through the treatment of refugees as inpatients using WGS-based data generated by our standard prospective surveillance procedures and epidemiological data. The application of cgMLST data for infection control in our institution is always combined with in depth epidemiological investigation [14].

In our study WGS-data demonstrated that the respective isolates were genetically heterogeneous and further revealed no definite transmission of RP isolates to other patients. Consequently, we omitted pre-emptive isolation for RP. Furthermore, whole genome sequencing-based typing did not show any evidence for nosocomial transmission from RP to other patients. Standard hygiene measures successfully prevented the transmission of RP isolates to other patients and, as a result, the introduction into our institution. We can therefore conclude that MDRO do not pose the risk of being introduced into health care facilities if screening procedures and standard hygiene measures are applied. This supports MDRO carried by patients with a refugee background do not pose any particular health threat to European citizens.

We were able to confirm the findings of other study groups that showed a higher prevalence of MDRO in the evaluated patient group in comparison to NRP, although Reinheimer *et al*. showed a slightly lower occurrence of MRSA and higher prevalence of MRGN [7]. Possible reasons for these slight differences in the prevalence to other studies can lay in the situations or characteristic of refugees depending on the city and area where the study takes place, i.e. length of stay in Germany or housing structures. VRE prevalence was low in our study and is therefore of less concern in refugee patients.

In our study, we noticed a statistically significant difference when comparing RP and NRP for MRSA. In our institution, a general admission screening for MRSA is implemented, whereas patients are only screened for MRGN and VRE if risk factors are identified. We therefore do not have a control group for these MDRO and did not include data derived from the screening of patients with risk factors. In a study focusing on gynecological patients, RP patients were matched with a control group of NRP. A significant difference in the prevalence of MRSA as well as MDR-GNB was shown [20].

Investigating MRSA *spa* types can serve as an indicator of genetic relatedness among RP and NRP isolates. Here, we saw differences between isolates from RP and NRP. *Spa* types found in RP do also differ from *spa t*ypes found in the general German general population as well as in the German healthcare system [21, 22]. *Spa* type t127, which was found in 14.7% of refugee-MRSA isolates, was found in 2.35% of isolates registered on the RIDOM-*Spa* Server which collates and harmonizes *spa*-typing data from various geographic regions [23]. *Spa* types t223 and t304 also showed high occurrence with 14.7% and 8.85% in RP isolates respectively. These *spa* types are each found in 0.4% each in isolates registered on the RIDOM-*Spa* Server. Up to now, t127 is mainly known as a non-CC398 LA-MRSA in Mediterranean Europe [24]. t223 is described as belonging to the most common *spa t*ypes in healthy Jordanian populations [25, 26], which might leads to the probable occurrence in refugee patients coming from Middle East countries.

A limitation of our study is the fact that screening was not performed in all refugee patients. Whereas 90.5% of RP were screened for MRSA, only 58.9% and 50.6% of RP underwent screening for MDR-GNB and VRE, respectively. This might be due to difficulties in the immediate identification of patients as refugees upon admittance. In some cases identification occurred only later during the course of the hospital stay. Additionally, cultural differences hindered the successful implementation of screening. Cultural affiliations are recognized to be a challenge in the provision of healthcare to migrants [27].

At the beginning of the study, there were no official recommendations concerning the hygienic measures to be taken in the treatment of refugee patients besides one document by the European Center for Disease Control (ECDC) considering screening for MDR-GNB in RP due to a high prevalence in the countries of origin [28]. Official German recommendations recommend screening for MRSA and MDR-GNB for patients with previous contact to health care facilities in countries of high prevalence [29]. Due to language barriers previous contact to health care could not always be reliably asserted. For these reasons we decided like other institutions for a restrictive approach, which included pre-emptive isolation until colonization by MDRO was excluded. Later, the RKI published a recommendation, which included neither isolation nor screening for this patient group in initial registration facilities. However, in the case of hospital stay, screening for MRSA and carbapenemase producing enterobacteriacea was recommended if the patient had had contact to health care facilities in countries with a high prevalence of MDRO [19].

MDRO prevalence in other studies was high and *spa* types were different from those occurring in NRP [7, 9], fundamentally questioning this approach. Hence the RKI did re-evaluate their recommendation resulting in screening for MRSA in all RP and not only those with previous contact to health care facilities [30]. An ongoing discussion remains as to which extent refugees should be pre-emptively isolated or screened upon admission to a hospital. Whereas some authors strongly advise pre-emptive isolation [7, 31], others question the appropriateness of this procedure alongside universal screening a measure [32, 33]. In contrast to others, we did not find any strains carrying carbapenemases. Our study adds WGS data to the discussion showing no nosocomial transmission of MDRO from RP to NRP if standard hygiene measures are applied. Therefore multidrug-resistant organisms carried by RP are not of any concern for health institutions and systems.

## Supporting information

S1 Table Collection date and MLST-sequences types of isolates compared in Figs 1f–3. (XLSX)
